# Editorial: Brain-image Based Computation for Supporting Clinical Decision in Neurological and Psychiatric Disorders

**DOI:** 10.3389/fnins.2020.599523

**Published:** 2021-03-03

**Authors:** Lin Shi, Weidong Cai, Feng Feng

**Affiliations:** ^1^Department of Imaging and Interventional Radiology, The Chinese University of Hong Kong, Shatin, China; ^2^School of Computer Science, The University of Sydney, Darlington, WA, Australia; ^3^Department of Radiology, Peking Union Medical College Hospital (CAMS), Beijing, China

**Keywords:** neurological disease, psychiatric disease, magnetic resonance imaging, functional connectivity, structural connectivity, machine learning, radiomics

Fast-paced developments in imaging and computational technologies in recent years have fostered breakthroughs in the conception and practice of neurology, neurosurgery, and psychiatry. This Research Topic presents a collection of recent researches on computational solutions for multimodal neuroimaging in brain disorders.

Structural imaging and its quantitative computational metrics are critical in assisting neurological disease diagnosis and assessment. A contribution by Zhang et al. explored the location of infarct core identified in diffusion weighted imaging (DWI) to distinguish intracranial atherosclerotic stenosis (ICAS) from embolic occlusion with good accuracy, which could help determine the most appropriate treatment strategy in the acute stage. Guo et al. studied the scanner-related reproducibility of white matter hyperintensity (WMH) in T2-FLAIR, which is crucial in the assessment of various white matter diseases, especially demyelinating, and small vessel diseases. The results showed that the best reproducibility occurred when 3D FLAIR images were acquired in the same MRI scanner, while WMH quantification variability reached the highest when WMH quantified in 2D FLAIR was compared with 3D FLAIR. The selection of quantification tools also made a big difference in reproducibility.

Computational imaging biomarkers can be applied to the study of mechanisms or predicting therapeutic strategies. Zhang et al. analyzed pre- and post- treatment resting-state functional MRI (rs-fMRI) images for dysphagia patients, examining the effect of intermittent theta burst stimulation (iTBS) after continuous TBS (cTBS) using static and dynamic functional connectivity and suggested that iTBS could reverse the aftereffects induced by cTBS on the contralateral suprahyoid muscle cortex. Rs-fMRI was also analyzed using regional homogeneity (ReHo), which detected significant differences among the data acquired at different times in patients with Nasopharyngeal Carcinoma (NPC) but not in healthy controls, suggesting the ReHo features may be indicative in predicting neurocognitive dysfunction in NPC (Yang et al.). Analysis of brain EEG features during the cognitive task could complement imaging studies to detect the temporal sensitive brain activity on-the-fly. Kim et al. present an EEG study to investigate the functional brain changes in subjects with Internet gaming disorder (IGD). They found that lower frontal theta activity might be a biomarker to detect diminished cognitive control in IGD.

The microarchitecture of the white matter provides sensitive and early evidence of brain changes. In the study by Liang et al., regional voxel-based group differences were found in inter-voxel diffusivity in T2 diabetes mellitus (DM) patients when compared with normal controls. These diffusion features were associated with T2DM risk factors. It is well-known that hippocampus volume asymmetry is an important visual clue for the detection of hippocampus sclerosis, which supports the proposition that white matter connectivity may also present as asymmetric. This is well-studied in a contribution by Zhao et al. using TBSS on diffusion tensor imaging (DTI) data. They found that the pattern of white matter asymmetry is distinct in TLE patients with left and right hippocampus sclerosis. In Jang et al., the authors investigated the diffusion metrics in MRI of the corticoreticulospinal tract (CRT), and analyzed the quantitative difference between mild traumatic brain injury (mTBI) patients with and without whiplash, and indicated that whiplash might be related to more severe axonal injuries in mTBI patients. The DTI data acquired in ADNI was employed in Liu et al. to develop a general framework that integrates deep learning, feature selection, causal inference, and genetic-imaging data analysis for predicting and understanding Alzheimer's Disease (AD).

Psychiatric diseases are usually reported with negative and inconsistent structural findings, but it is likely that statistical changes exist when microstructural or functional information is being studied. Fu et al. studied patients with schizophrenia and reported that IL-10, a regulatory cytokine assessed in venous blood, is associated with white matter integrity changes in DTI, using tract-based spatial statistics (TBSS) analysis. Another study in this Research Topic by Ding et al. explored the functional connectivity changes in schizophrenia. In this study global-brain FC was used on rs-fMRI data, indicating that schizophrenia patients and unaffected siblings shared enhanced GFC in the left superior frontal gyrus compared with normal controls. These findings separated those with schizophrenia and their siblings from normal controls. Lidaka et al. conducted a study of functional connectivity using rs-fMRI on a large sample (*n* = 626) of Autism Spectrum Disorder (ASD) and normal controls and found that patients with ASD exhibited thalamo-cortical hyperconnectivity and amygdala-cortical hypoconnectivity, which might be a potential neuroimaging biomarker of ASD. In another study of ASD, Zhou et al. propose a regularized learning framework to generate a high-order functional connectivity network (HoFCN) with high reliability and applied it to the identification of mild cognitive decline (MCI) and ASD from normal controls. This mathematics-based development was proven to be effective in making disease-related functional MRI changes in patients. Magnetic Resonance Spectroscopy (MRS) is a powerful tool and can be applied to the study of intracranial chemical composition with magnetic pulse sequences. Wang et al. introduced their work on using the MRS to quantify Gamma-Aminobutyric Acid (GABA) levels in women with depression and anxiety. In the postmenopausal stage, lower GABA levels were found in the depression group compared with the anxiety group, and both groups were lower than normal controls.

The development of computational methods, i.e., machine learning and radiomics, provides powerful tools to support systematic investigation for brain research. In Liu et al., the authors introduced their work on developing a machine learning prediction model based on radiomics features derived from MRI, to better characterize and distinguish neonatal acute bilirubin encephalopathy (ABE) from normal myelination in T1W MRI with high accuracy. The contribution by Zheng et al. also discusses how machine learning (namely support vector machine, SVM) was performed on the quantitative volumetry results of patients diagnosed with AD and vascular dementia (VaD). They generated a classification model with high accuracy, which indicated that there could be distinct brain atrophy patterns between these two major types of dementia.

The research efforts documented in these publications contribute to and further computational neuroradiology, and is particularly enlightening on potential future clinical applications.

## Author Contributions

All authors listed have made a substantial, direct and intellectual contribution to the work, and approved it for publication.

## Conflict of Interest

The authors declare that the research was conducted in the absence of any commercial or financial relationships that could be construed as a potential conflict of interest.

